# Age-related decrements in dual-task performance: Comparison of different mobility and cognitive tasks. A cross sectional study

**DOI:** 10.1371/journal.pone.0181698

**Published:** 2017-07-21

**Authors:** Paolo Riccardo Brustio, Daniele Magistro, Massimiliano Zecca, Emanuela Rabaglietti, Monica Emma Liubicich

**Affiliations:** 1 Department of Psychology, University of Torino, Torino, Italy; 2 NeuroMuscularFunction | Research Group, School of Exercise and Sport Sciences, Department of Medical Sciences, University of Torino, Torino, Italy; 3 School of Sport, Exercise, and Health Sciences, Loughborough University, Loughborough, United Kingdom; 4 NCSEM – National Centre for Sport and Exercise Medicine, Loughborough, United Kingdom; 5 Wolfson School of Mechanical, Electrical and Manufacturing Engineering, Loughborough University, Loughborough, United Kingdom; 6 SUISM, University of Torino, Torino, Italy; Universidad Pablo de Olavide, SPAIN

## Abstract

This cross-sectional study investigated the age-related differences in dual-task performance both in mobility and cognitive tasks and the additive dual-task costs in a sample of older, middle-aged and young adults. 74 older adults (M = 72.63±5.57 years), 58 middle-aged adults (M = 46.69±4.68 years) and 63 young adults (M = 25.34±3.00 years) participated in the study. Participants performed different mobility and subtraction tasks under both single- and dual-task conditions. Linear regressions, repeated-measures and one-way analyses of covariance were used, The results showed: significant effects of the age on the dual and mobility tasks (*p*<0.05) and differences among the age-groups in the combined dual-task costs (*p*<0.05); significant decreases in mobility performance under dual-task conditions in all groups (*p*<0.05) and a decrease in cognitive performance in the older group (*p*<0.05). Dual-task activity affected mobility and cognitive performance, especially in older adults who showed a higher dual-task cost, suggesting that dual-tasks activities are affected by the age and consequently also mobility and cognitive tasks are negatively influenced.

## Introduction

In everyday life, the mobility, defined as the ability to independently move around the environment [1], often requires the simultaneous performance of multiple cognitive or motor tasks. In this situation, the successful performance of a simultaneous task is essential for the independence of older adults, and it may be difficult [2, 3], due to the decline of physical [4–6] and cognitive [7] function (i.e. executive functions) during the aging process.

The dual-task paradigm has been used to investigate the performance of simultaneous tasks in older adults. Dual-task conditions involve attention and executive function processes [8–11] and typically require performing a primary task while simultaneously carrying out a concurrent secondary task [12, 13]. Previous studies used various sensorimotor primary tasks, such as balance or walking tasks, and a secondary task, such as cognitive tasks (e.g., reaction time, discrimination and decision-making, working memory tasks) or manual tasks (e.g., upper manipulation of an object) [8, 14–16]. These results underlined a possible cognitive-motor interference under dual-task conditions, particularly in older adults compared to young adults, that led to a decline in the mobility performance and secondary task, or both [17], depending on the individual capability and task type [3, 15, 17].

Previous studies emphasized the role of the cognitive system during mobility performance and the dependency of automatic and high-level cognitive processes [8, 14], especially in advancing age. Indeed, mobility performance is a multi-dimensional process and requires a high level of motor control and cognitive flexibility [1, 18], in order to pay attention to various external features and stimuli [1, 19]. For example, with a complex postural balance task older adults are not able to perform the concurrent activity as young adults, suggesting that with age the stability of posture becomes less automatic and more controlled by the cognitive resources [20]. Yet, studies on gait pattern emphasized the influence of the executive function during the dual task performance [8, 21] and observed the interference with gait (reduced gait speed and increased variability [2, 10, 22–25]). Moreover, focusing on the impact of mobility performance on cognitive tasks, older adults are not able to perform the cognitive secondary tasks as well as young adults. For example, using a serial subtraction of 3 and 7, Srygley and colleagues [11] showed that a walking task might alter the cognitive performance in term of number of mistakes among both healthy young and older adults, but the decrease of the cognitive performance was larger in older adults. The above findings suggested that mobility performance affects cognitive performance with age, especially when the cognitive task is difficult, probably due to the decline in executive function observed in old age [11]. Taken together, the decreases in both mobility and cognitive performance, interpreted as dual-task cost (e.g., the reduction of the performance under dual-task conditions relative to the single-task condition) [26], may lead to an impairment of everyday life activities and therefore to a mobility limitation [27] or to an increased risk of falling [15]. The above findings have underlined that dual-task performance may be an useful clinical test of the functional decline and frailty in older adults, and consequently it can identify changes at an earlier stage for allowing a quick intervention to prevent adverse outcomes [28].

Recently, Plummer and colleagues [27, 29] proposed a new framework for the cognitive-motor interference measurement considering both mobility and cognitive tasks and consequently taking into account the interactions between the two tasks as indicator of task abilities. This approach allowed to investigate the attentional strategy and potential trade-offs [29]. Indeed, the measurement of only one parameter of the performance, generally the mobility task, could lead to misleading conclusions about the effects of dual-task performance especially in rehabilitation setting. Again, this assessment may help not to confuse the results with the real priorities [2, 30]. Furthermore, as per the task prioritization model [29, 31], during competition for attention resources an individual can decide which of the two tasks dedicate more attention for avoiding dangers and obtaining a better performance and consequently, this different strategy could influence the direction and magnitude of dual task evaluation [29].

However, to the best of our knowledge, only few studies [10, 32, 33] have investigated the effect of mobility performance on cognitive tasks.

Furthermore, previous studies [e.g. 22, 34] examined dual-task effects on mobility performance in aging people, but as noted in a meta-analysis on the topic [14], few studies have investigated the age-related difference in dual-task performance with a middle-aged group. This evaluation may be useful to explore and to better understand the possible onset of difficulty in dual-task performance during the life span [34]. The comprehension of the cognitive-motor interferences that arise in dual-task performance, considering both motor and cognitive costs in older adults, may be useful to design interventions aimed to improve the cognitive and motor skills involved in these situations. Moreover, this knowledge may be an indicator of current and future aging-related declines in older adults (e.g., possible fall risk). Additionally, the inclusion of a middle-aged people could provide a better understanding of the effects of the age on activities of daily life that integrate motor and cognitive interactions.

Accordingly, the purpose of the current study was to use different mobility and secondary cognitive tasks to investigate the changes occurring during dual-task performance in a relatively large sample of young, middle-aged and older adults. Therefore, the specific aims of the study were 1) to determine age-related differences in dual-task performance both in mobility and cognitive tasks by comparing the performance of young, middle-aged and older adults and 2) to investigate the additive, age-related, dual-task costs considering both the mobility and cognitive costs.

Our main hypothesis is that it could be possible to observe age-related differences in dual-task performance due to the changes in physical and cognitive functions during the life span, in particular in the executive functions. Consequently, a decreasing trend in dual-task performance with age increasing should be observed. Moreover, the older people should show a worse performance in both mobility and cognitive tasks compared to the younger people. Furthermore, considering both the mobility and the cognitive dual-task cost, we expect to observe an increase in combined dual-task costs with age advancing, presumably due to insufficient capabilities of the central processing [14, 35]. Therefore, it is important to investigate different motor activities, such as mobility, in the presence of additional attention demanding cognitive and motor tasks. This could improve our understanding of age-related differences in dual task performances, which will be clinically significant to guide our future actions for improving the health and socio-economic consequences of the ageing process, including decline in cognitive and physical abilities, life skills, and in overall quality of life.

## Materials and methods

### Participants

One hundred ninety-five subjects participated in the study: 63 younger subjects between the age of 20 and 35 years (mean age = 25.34 ± 3.00 years; 30 females), 58 middle-aged subjects between the age of 40 and 55 years (mean age = 46.69 ± 4.68 years; 37 females), and 74 older subjects between the age of 65 and 85 years (mean age = 72.63 ± 5.57 years; 51 females). All of the participants were enrolled through public advertisements in the community. The inclusion criteria were as follows: the ability to live independently, the ability to walk without an assistance device (e.g., cane or walker), and a mini-mental status examination (MMSE) score ≥ 24 or higher in older subjects. Participants were excluded if they presented certain medical conditions, such as an acute disease (e.g., myocardial infarction, coronary bypass surgery) or a chronic disease (e.g., Alzheimer’s disease, Parkinson’s disease) or musculoskeletal conditions (e.g., orthopaedic impairments, upper or lower extremity fracture in the past 6 months) affecting mobility or balance and if they were participating in another study. The participants did not receive any incentive to participate. All participants were informed that participation in the study was voluntary and confidential, and they provided written informed consent prior to the data collection. The Ethical Committee of the University of Torino approved the study. The study was conducted during the first quarter of 2014.

### Mobility tasks

The mobility tasks consisted of the 10-meter Walking Test [36], the Timed Up and Go test [37], and the Four Square Step test [38].

The 10-meter Walking Test (10WT) requires walking for 10 m without assistance at a comfortable, normal pace. This performance time was measured when the subjects crossed the 2-m mark and the 8-m mark [39]. The 2-m mark and the 8-m mark, considered as warm-up and deceleration phases, respectively, were not included in the calculation [40].

The Timed Up and Go (TUG) test measures the time taken by a subject to stand up from an arm chair, walk a distance of 3 meters, turn, walk back to the chair and sit down [37].

The Four Square Step (FSS) test measures the time taken by a subject to rapidly change direction while he or she is stepping forward, backward and sideways in a predetermined sequence over four walking sticks placed in a cross configuration on the ground [38].

The timed performance, in seconds, of each mobility task was measured with a stopwatch, recorded and used for future analyses.

### Cognitive tasks

The cognitive tasks were arithmetic tasks and consisted of a serial subtraction of 3 and 7 from a random number between 80 and 99 [11, 23, 41, 42]. These tasks involved working memory function [11]; therefore, they were directly linked with executive functions [43]. According to Srygley and colleagues [11], the two arithmetic tasks were chosen to provide different levels of task difficulty.

The single tasks were performed in a seated position for 30 s, and the performance was individually adjusted for each mobility task combination to be equivalent to the dual-task time [32, 33]. During the single- and dual-task conditions, the total number of subtractions for each cognitive task was counted, and every mistake was noted. We quantified the performance of the cognitive task considering both percentage of accuracy and response rate. According to Hall and colleagues [10] the average Correct Response Rate (CCR = response rate per second × percent of accuracy) was computed both for single- and dual-task performance.

### Procedures

After the screening for inclusion criteria, participants filled a self-report questionnaire concerning socio-demographic characteristics. Afterwards, the participants attended a single data collection session and were tested under both the single- and dual-task conditions, including the following:

1. single mobility tasks (10MW test, TUG test, and FSS test);
2. single cognitive tasks (serial subtraction of 3 and 7);
3. dual-task condition: mobility tasks and serial subtraction of 3;
4. dual-task condition: mobility tasks and serial subtraction of 7.

To reduce possible fatigue effects, a rest period was given after each single- and dual-task condition, dependent on each subject [32]. The subjects were instructed to perform each mobility task at their self-selected comfortable pace.

The order of the tasks for each participant was randomly chosen using a random number generator to avoid performance bias in the mobility and cognitive tasks. No instructions were given regarding which task to prioritize during the dual-task condition, in order to establish an ecological situation similar to real life [41].

### Data analysis

To quantify the mobility dual-task costs (DTCs_mobility tasks_) in each subject for all combination tasks, we used the following formula: DTCs_mobility tasks_ = 100 × [(dual-task mobility score—single-task mobility score)/single-task mobility score] [10]. Specifically, the single-task mobility score indicated the performance time in the 10MW test, the TUG test, and the FSS test, while the dual-task mobility score indicated the performance time in the 10MW test, TUG test, FSS test during serial subtractions of 3 and 7, respectively. Alternatively, to focus on cognitive performances, we calculated the cognitive dual-task costs (DTCs_cognitive tasks_) using the following formula: DTCs_cognitive tasks_ = 100 × [(single CCR score—dual-task CCR score)/single CCR score]. The single-task CCR score indicates the average correct response rate in the seated position, while the dual-task cognitive score indicates the average correct response rate during the 10MW test, the TUG test, and the FSS test. We computed the dual-task cost for mobility and cognitive performances separately, in order to obtain comparable results. Positive values indicate a worse performance during the dual-task conditions, while negative values indicate a better performance. To quantify the dual-task ability, we calculated the combined mean DTC (mDCT) during dual-task performance in all combination tasks using the following formula: mDTC [%] = [DTC_mobility task_ + DTC_cognitive task_]/2 [2, 35]. Here too, positive mDTC values indicate a worse performance in the dual-task cost condition, while negative mDTC values indicate a better performance.

A series of multiple regressions was run to predict mDT in the different combination tasks from age and gender (males and females) separately. Moreover, in order to explore dual-task performance, a series of multiple regressions was separately run for all combination tasks of both mobility and cognitive tasks.

Controlling for gender, one-way analyses of covariance (ANCOVAs) were used to assess the differences in mDTC among the groups. A post hoc analysis with a Bonferroni adjustment was computed to identify the age comparisons that were statistically significant. Finally, using gender as a covariate, a series of ANCOVA with a between-factor of Age (young, middle-aged and older adults) and a within-factor of Task Condition was used for determining the age-related differences in mobility task performance (single-task, dual-task condition subtraction 3, dual-task condition subtraction 7) and the average correct response rate in cognitive tasks (seated position and subtraction 3 during each mobility task; seated position and subtraction 7 during each mobility task). A post hoc analysis with a Bonferroni adjustment was computed to identify the age comparisons and Age × Task that were statistically significant.

The significance level was set at *p* ≤ 0.05. The Statistical Package for Social Sciences (SPSS 20.0 for Windows) was used for all statistical analyses.

## Results

### Sample characteristic

The socio-demographic characteristics of the participants are summarized in Table 1. The mean body mass index was approximately 22 ± 3 kg m^-2^ for younger subjects, 24 ± 4 kg m^-2^ for middle-aged subjects and 26 ± 4 kg m^-2^ for older adults. Generally, older adults presented a lower education (M = 8 ± 3 years) compared to young (M = 18 ± 2 years) and middle-aged women (M = 13 ± 4 years.

**Table 1:** Socio-demographic characteristics of subjects.

	Age Groups		
Characteristics	Young	Middle-aged	Older
Age (years)	25.34 ± 3.00	46.69 ± 4.68	72.63 ± 5.57
Height (m)	1.72 ± 7.85	1.66 ± 9.22	1.63 ± 8.52
Weight (kg)	65 ± 12	67 ± 13	71 ± 12
BMI (kg m^-2^)	22 ± 3	24 ± 4	26 ± 4
Education years (years)	18 ± 2	13 ± 4	8 ± 3
Gender, n (%)			
Female	30 (47.6)	21 (36.2)	23 (29.9)
Male	33 (52.4)	37 (63.8)	51 (43.2)

*Notes*: Data presented as mean and standard deviation or percentage. BMI: Body Mass Index

### Age influence

Table 2 summarizes the results of regression analysis for combined mean dual-task cost, mobility performance, and cognitive performance. Positive significant influence of age was observed in mDTC 10WT and subtraction 7, mDTC TUG and subtraction 3 and mDTC TUG and subtraction 7. Regarding mobility performance in single and dual-task conditions, the regression outcomes revealed a positive influence of age in all proposed tests, indicating a worse performance in advancing age as hypothesised (for more detail see Table 2). Conversely, the regression outcomes of the cognitive performance in the proposed tasks showed a negative influence of age in all dual-task tests, but not in the majority of the sitting tasks (Table 2).

**Table 2:** Multiple regression model for combined mean dual-task cost, motor and cognitive performances.

outcomes		Independent Variables						Model	
		Age			Gender				
		B	SE B	ß	B	SE B	ß	R^2^	F
Combined mean dual-task cost (mDTC)	10WT and subtraction 3	0.004	0.002	0.188*	-0.017	0.064	-0.019	0.035	3.218*
	10WT and subtraction 7	0.004	0.002	0.144	0.084	0.081	-0.080	0.043	1.946*
	TUG and subtraction 3	0.003	0.001	0.283***	0.035	0.034	0.073	0.091	8.977***
	TUG and subtraction 7	0.004	0.001	0.245**	0.076	0.045	0.125	0.084	7.835**
	FSST and subtraction 3	0.001	0.001	0.091	-0.020	0.045	-0.034	0.009	0.773
	FSST and subtraction 7	0.003	0.001	0.156*	-0.037	0.055	-0.052	0.025	2.130
Motor task	10WT (s)	0.023	0.003	0.475***	0.102	0.131	0.050	0.237	29.760***
	10WT and S3 (s)	0.047	0.006	0.494***	0.186	0.249	0.048	0.255	32.917***
	10WT and S7 (s)	0.054	0.007	0.509***	0.268	0.276	0.061	0.275	36.399***
	TUG (s)	0.049	0.005	0.590***	0.262	0.203	0.075	0.371	56.563***
	TUG and S3 (s)	0.076	0.008	0.586***	0.567	0.313	0.105	0.378	58.337***
	TUG and S7 (s)	0.079	0.008	0.558***	0.453	0.352	0.077	0.334	48.103***
	FSS (s)	0.076	0.006	0.675***	0.195	0.250	0.042	0.468	84.313***
	FSS and S3 (s)	0.101	0.010	0.595***	0.641	0.407	0.091	0.338	59.517***
	FSS and S7 (s)	0.116	0.012	0.577***	0.120	0.503	0.014	0.337	48.732***
Cognitive task S3	Sitting (%)	-0.376	0.118	-0.210**	-25.724	4.929	-0.345***	0.191	22.609***
	10WT (%)	-0.415	0.109	-0.256***	-17.618	4.558	-0.261***	0.159	18.087***
	Sitting (%)	-0.317	0.095	-0.213**	-25.782	3.946	-0.415***	0.252	32.271***
	TUG (%)	-0.462	0.082	-0.345***	-20.803	3.411	-0.373***	0.307	42.581***
	Sitting (%)	-0,390	0.092	-0.265**	-25.636	3.797	-0.420***	0.287	38.471***
	FSS (%)	-0.342	0.073	-0.302***	-15.518	3.027	-0.329***	0.237	29.889***
Cognitive task S7	Sitting (%)	-0.208	0.081	-0.175*	-15.542	3.356	-0.314***	0.150	16.936***
	10WT (%)	-0.295	0.079	-0.254***	-11.725	3.286	-0.242**	0.146	16.473***
	Sitting (%)	-0.150	0.083	-0.122	-17.848	3.455	-0.349***	0.153	17.362***
	TUG (%)	-0.197	0.052	-0.245***	-110.881	20.177	-0.355***	0.219	26.899***
	Sitting (%)	-0.137	0.064	-0.141*	-15.665	2.663	-0.389***	0.196	22.673***
	FSS (%)	-0.187	0.053	-0.239**	-8.968	20.186	-0.274***	0.157	17.872***

*Notes*: mDTC, combined mean dual-task cost; 10WT, 10-meter Walking Test; TUG, Timed Up and Go test; FSS, Four Square Step test; S3, subtraction of three; S7, subtraction of seven; 10WT, 10-meter Walking Test; TUG, Timed Up and Go test; FSS, Four Square Step test.

* indicates *p* < 0.05;

** indicates *p* < 0.01;

*** indicates *p* < 0.001.

### Group differences

#### Combined mean DTC

**Fig 1:**
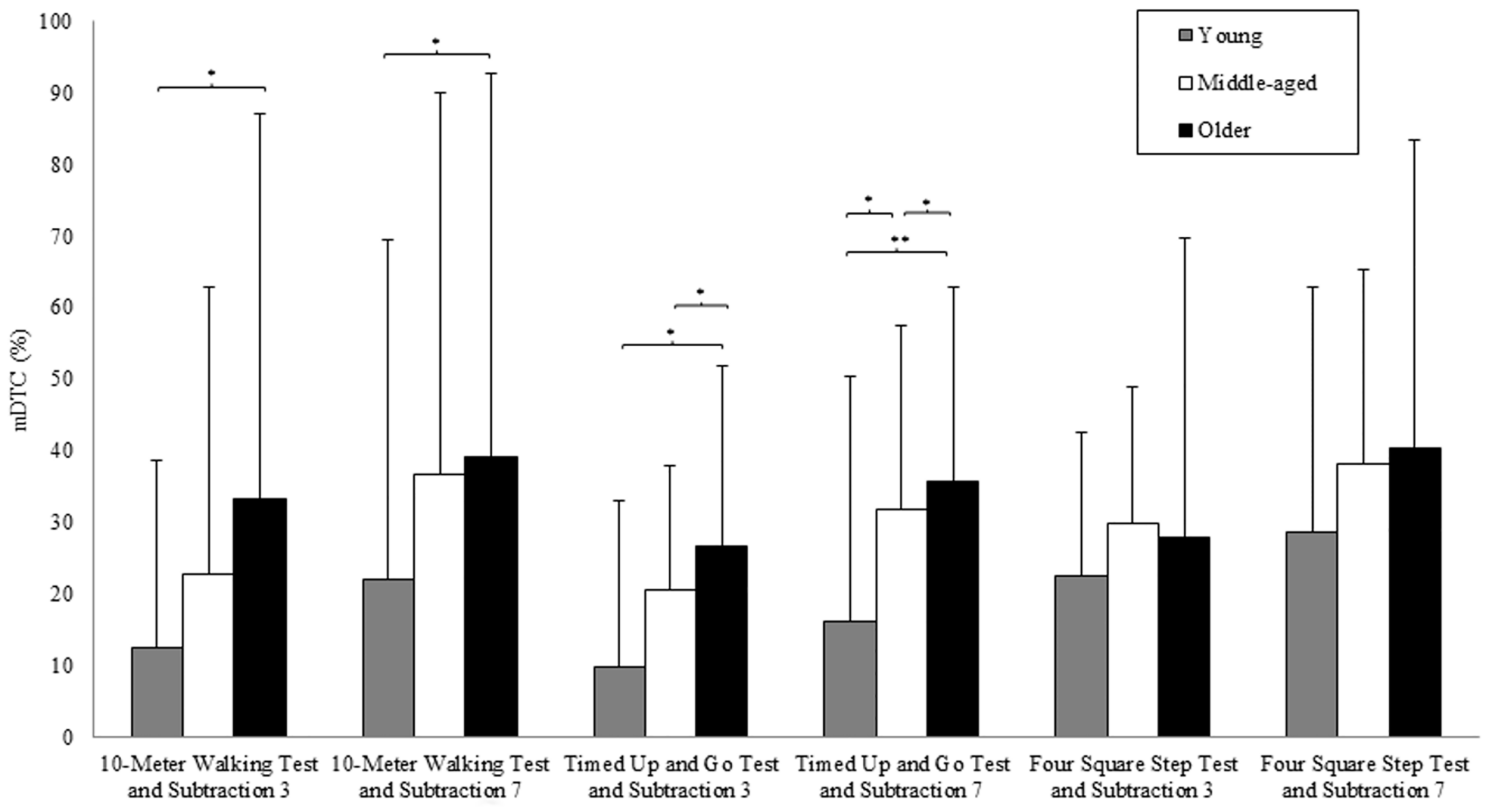
Combined mDTC. Mean dual-task costs (mDTCs) for the combined mobility and cognitive tasks. Each bar represents the average score for young (gray), middle-aged (white) and older subjects (black) in the different mobility tasks. The columns represent the means, and the error brackets represent the pertinent standard error. * indicates *p*< 0.05; ** indicates *p*< 0.01.

Fig 1 represents the combined mDTC values separately in young, middle-aged and older subjects. The combined mDTC values clustered in two ranges among the three groups: approximately 10–40% for most mobility tasks with subtraction of 3, and approximately 20–40% for the mobility tasks with subtraction of 7. Generally, older subjects presented higher mDTC values compared to middle-aged and young subjects in most combination tasks.

According to the above observations, the ANCOVA yielded a significant difference for Age in the mDTC of the 10MW test and TUG test with serial subtraction of 3 and 7 and in the FSS test with serial subtraction of 7 (Table 3).

**Table 3:** ANCOVAs and post hoc results for the mDTCs.

Task combination	Age	Post hoc		
		p 1	p 2	p 3
mDTC 10WT and subtraction 3	F_2,183_ = 3.943*	*	n.s.	n.s.
mDTC 10WT and subtraction 7	F_2,191_ = 3.943*	*	n.s.	n.s.
mDTC TUG and subtraction 3	F_2,181_ = 8.505***	*	*	n.s.
mDTC TUG and subtraction 7	F_2,172_ = 6.293**	**	*	*
mDTC FSST and subtraction 3	F_2,181_ = 1.120^n.s^	n.s.	n.s.	n.s.
mDTC FSST and subtraction 7	F_2,172_ = 1.906^n.s^	n.s.	n.s.	n.s.

*Notes*: mDTC, combined mean dual-task cost; 10WT, 10-meter Walking Test; TUG, Timed Up and Go test; FSS, Four Square Step test; p 1, post hoc results for comparing the young and older groups; p 2, post hoc results for comparing the young and middle-aged groups; p3, post hoc results for comparing the middle-aged and older groups.

* indicates *p* < 0.05;

** indicates *p* < 0.01.

#### Mobility task performance

Table 4 summarizes the effects of single- and dual-task conditions on mobility tasks separately for young, middle-aged and older subjects, as well as the ANCOVA outcomes and the post hoc results. In general, older subjects did not perform as well as young and middle-aged groups in the single- and dual-task conditions. Yet, the performance time in all mobility measures increased among the different age groups from single to dual tasks.

**Table 4:** ANCOVAs and post hoc results of the mobility performance tests.

Task	Young M (SD)	Middle-aged M (SD)	Older M (SD)	Age	Task	Age × Task	Post hoc Task			Post hoc Age		
							t1	t2	t3	p1	p2	p3
10WT (s)	4.07 (0.59)	4.40 (0.64)	5.09 (1.23)	F_2,191_ = 29.931***	F_2,382_ = 48.297***	F_4,382_ = 9.387***	***	***	***	**	*	**
10WT and S3 (s)	4.69 (0.82)	5.46 (1.14)	6.70 (2.46)									
10WT and S7 (s)	4.98 (0.98)	5.98 (1.38)	7.42 (2.27)									
TUG (s)	6.66 (0.91)	7.05 (0.87)	8.83 (1.98)	F_2,191_ = 47.843***	F_2,382_ = 69.945***	F_4,382_ = 9.830***	***	***	***	***	n.s.	***
TUG and S3 (s)	7.52 (1.25)	8.26 (1.25)	10.97 (3.12)									
TUG and S7 (s)	8.09 (1.49)	8.94 (1.62)	11.66 (3.38)									
FSS (s)	7.53 (1.01)	8.86 (1.35)	11.09 (2.35)	F_2,191_ = 61.686***	F_2,382_ = 107.377***	F_4,382_ = 6.490**	***	***	***	***	***	***
FSS and S3 (s)	9.54 (1.98)	11.79 (2.18)	14.37 (3.70)									
FSS and S7 (s)	10.27 (2.08)	12.89 (2.52)	15.86 (4.66)									

*Notes*: M, mean; SD, standard deviation; 10WT, 10-meter Walking Test; TUG, Timed Up and Go test; FSS, Four Square Step test; S3, subtraction of three; S7, subtraction of seven; t1, post hoc results for comparing single and dual-task subtraction 3; t2 post hoc results for comparing single and dual task subtraction 7; t3 post hoc results for comparing dual task subtraction 3 and dual-task subtraction 7; p1, post hoc results for comparing the young and older groups; p2, post hoc results for comparing the young and middle-aged groups; p3, post hoc results for comparing the middle-aged and older groups.

* indicates *p* < 0.05;

** indicates *p* < 0.01;

*** indicates *p* < 0.001.

Specifically, Table 2 highlights the larger performance time of older subjects in all conditions of the 10MW test in comparison with young and middle-aged groups. These findings are reflected in the ANCOVA outcomes by significant effects of Age, Task Condition, and Age × Task Condition interaction (Table 4). Moreover, the post hoc for Age results yielded a significant difference among the Task Condition and Age groups (Table 4). The Table 5 highlights the post hoc analysis for the significant Age × Task Condition interaction.

**Table 5:** ANCOVAs and post hoc results of interaction Age × Task.

			t 1	t 2	t 3
Single-task	10WT	F_2,191_ = 21.020***	***	***	***
	TUG	F_2,191_ = 43.395***	***	n.s.	***
	FSS	F_2,191_ = 70.116***	***	***	***
Dual-task subtraction 3	10WT	F_2,191_ = 24.712***	***	n.s.	***
	TUG	F_2,191_ = 45.221***	***	n.s.	***
	FSS	F_2,191_ = 46.333***	***	***	***
Dual-task subtraction 7	10WT	F_2,191_ = 25.759***	***	n.s.	***
	TUG	F_2,191_ = 38.184***	***	***	n.s.
	FSS	F_2,191_ = 43.944***	***	***	***

*Notes*: M 10WT, 10-meter Walking Test; TUG, Timed Up and Go test; FSS, Four Square Step test; S3, subtraction of three; S7, subtraction of seven; t1, post hoc results for comparing single and dual-task subtraction 3; t2 post hoc results for comparing single and dual task subtraction 7; t3 post hoc results for comparing dual task subtraction 3 and dual-task subtraction 7.

* indicates *p* < 0.05;

** indicates *p* < 0.01;

*** indicates *p* < 0.001.

Concerning the performance in the TUG test, young and middle-aged subjects showed similar results both in single- and dual-task conditions. On the contrary, the performance of the single- to dual-task conditions of older subjects was more pronounced. Indeed, older adults performed the TUG more slowly in the single and the dual-task conditions compared to the other groups. According to the above findings, the ANCOVA results yielded significant effects for Age, Task Condition, and Age × Task Condition interaction (Table 4). The post hoc analysis revealed significant differences when comparing the young and older groups and the middle-age and older groups (Table 2). The Table 3 highlights the post hoc analysis for the significant Age × Task Condition interaction.

Lastly, in the FSS test, older adults generally performed worse than young and middle-aged groups in all single- and dual-task conditions. The ANCOVA results showed significant effects for Age, Task Condition, and Age × Task Condition interaction (Table 4). The post hoc tests showed significant differences when comparing the young and older groups and the middle-aged and older groups (Table 4). The Table 5 highlights the post hoc analysis for the significant Age × Task Condition interaction.

#### Cognitive task performance

Table 6 provides average correct response rate (CCR) in the cognitive tasks and the results of the analyses of covariance (ANCOVA).

**Table 6:** ANCOVAs and post hoc results of the cognitive performance tests.

Task		Young M (SD)	Middle-aged M (SD)	Older M (SD)	Age	Task	Age * Task	Post hoc Task		
								t1	t2	t1
S3	Sitting (%)	72.20 (31.64)	67.20 (36.55)	54.48 (38.66)	F_2,191_ = 5.016**	F_1,191_ = 5.322*	F_2,191_ = 0.504^n.s.^	**	^n.s.^	^n.s.^
	10WT (%)	71.00 (29.24)	61.95 (34.29)	50.20 (33.13)						
	Sitting (%)	68.87 (27.12)	63.21 (28.15)	51.56 (32.66)	F_2,191_ = 9.737***	F_1,191_ = 28.165***	F_2,191_ = 2.343 ^n.s.^	***	^n.s.^	**
	TUG (%)	61.54 (25.64)	52.33 (25.64)	36.57 (24.97)						
	Sitting (%)	68.11 (29.51)	59.94 (27.86)	46.87 (29.96)	F_2,191_ = 10.330***	F_1,191_ = 68.547***	F_2,191_ = 0.031 ^n.s.^	***	^n.s.^	**
	FSS (%)	50.61 (21.81)	44.62 (21.69)	32.53 (22.10)						
S7	Sitting (%)	40.67 (23.77)	40.89 (23.31)	30.93 (24.26)	F_2,191_ = 8.884**	F_1,191_ = 6.238**	F_2,191_ = 1.305 ^n.s.^	*	^n.s.^	**
	10WT (%)	38.18 (22.90)	33.95 (28.02)	22.55 (17.66)						
	Sitting (%)	37.70 (26.12)	37.94 (22.87)	28.55 (24.96)	F_2,191_ = 5.086**	F_1,191_ = 33.323***	F_2,191_ = 0.744 ^n.s.^	*	^n.s.^	*
	TUG (%)	28.98 (16.25)	25.73 (16.87)	17.45 (14.24)						
	Sitting (%)	32.69 (19.75)	34.00 (20.90)	24.39 (17.67)	F_2,191_ = 5.841**	F_1,191_ = 46.642***	F_2,191_ = 1.634^n.s.^	*	^n.s.^	**
	FSS (%)	25.70 (17.67)	22.57 (15.60)	15.59 (13.34)						

*Notes*: M, mean; SD, standard deviation; S3, subtraction of three; S7, subtraction of seven; 10WT, 10-meter Walking Test; TUG, Timed Up and Go test; FSS, Four Square Step test; p1, post hoc results for comparing the young and older groups; p2, post hoc results for comparing the young and middle-aged groups; p 3, post hoc results for comparing the middle-aged and older groups.

* indicates *p* < 0.05;

** indicates *p* < 0.01;

*** indicates *p* < 0.001

Generally, older adults were less accurate in both single and dual-task conditions than the young and middle-aged groups. The CCR yielded significant effects for Age and Task Condition while performing all mobility tasks with serial subtraction of 7 when compared to serial subtraction of 7 in seated position. Post hoc tests for Age elicited significant differences in serial subtraction of 3 and 7 when comparing the young and older groups and between the middle-aged and older groups while performing the 10MW test, TUG test and FSS test (Table 6).

## Discussion

This study aimed to investigate the age-related effects and differences in dual-task performance on mobility and cognitive tasks, and the combined dual-task costs considering both the mobility and cognitive performance in a sample of young, middle-aged and older adults. For this purpose, we focused on the changes that occurred in the dual-task performance both in different mobility tasks and cognitive tasks by comparing the performances of young, middle-aged and older adults and on the combined dual-task cost in mobility and cognitive performance.

Considering the first aim of the study, we hypothesized that a decrease in mobility and cognitive performance under the dual-task condition would be observed, with a worse performance in older adults. Consistent with our hypothesis, we observed a significant effect of the cognitive tasks on the three age groups with a larger decrease in mobility performance in older adults. Additionally, we observed a decreasing trend in mobility performance in the three groups when multicomponent activities were involved, such as sit-to-stand movement, initiation of stepping, turning movement or stepping forward, backward and sideways with a shift of the center of gravity to maintain a stable posture. In fact, during the TUG and FSS, we observed a larger decrease in the mobility performance in all groups, and this decrease was larger in older adults compared to young and middle-aged adults both during the serial subtraction of 3 and 7. The changes in mobility performance shown in our study are similar to the changes observed in previous works where a similar cognitive task was used [22–24, 44]. Holland and colleagues [22] reported similar decreasing trends in young, middle-aged and older adults during a walking task, concluding that dual-task performance may have a negative effect on walking, leading to a greater risk of falling in aging people. Similarly, we observed that older adults’ performances were worse in the dual-task condition in each mobility task compared with the single task, presumably due to the insufficient capabilities of central processing [14, 35]. In addition, we noticed that the cognitive task complexity can differentially lead to a performance decrease of the different mobility tasks. Indeed, our results indicated a larger decrease in mobility performances during serial subtraction of 7 than of 3. For example, during the TUG test, the mobility performance of older adults decreased by approximately 32% with serial subtraction of 7 and approximately 24% with serial subtraction of 3. Similar trends were observed also for the 10WT and FSS test. The execution of the two different tasks requires executive function processes [8–11]. Moreover, counting backward is directly linked with executive functions [43]. Thus, we could hypothesize that the serial subtraction of 7 involved more resources of executive function compared to the subtraction of 3. This might explain the larger decrease in mobility performance during the serial subtraction of 7. Conversely, we found that the response of young, middle-aged and older adults to cognitive loading in the different mobility tasks apparently depends on the difficulty level of the cognitive task [3, 11, 15].

Focusing on the impact of the mobility tasks on cognitive task performance, we observed that the motor performance might affect the cognitive task performance depending on the level of difficulty of the cognitive task. These differences might indicate that mobility tasks can affect cognitive performance depending on the complexity level of the performed cognitive task [11]. The results seem to suggest that an appropriate level of complexity could be specific to the population of interest and that, with a properly chosen complexity level, a positive and indicative finding is likely. For example, it might be more appropriate to use a simple task level for frail people, such as counting backwards by 3 and a more difficult level of secondary task might be more appropriate for healthy people, such as counting backwards by 7.

The second aim of the study was to investigate the additive age-related costs considering both the mobility and cognitive costs. We expected to observe an increase of combined dual-task cost in old age. Actually, we observed that the combined dual-task cost was larger for some mobility and cognitive task combinations in older adults, partially supporting our hypothesis. Specifically focusing on walking task performance, our results correlated with recent reviews [35] that calculated the combined dual-task costs in walking tasks from different studies. We found dual-task costs similar to the study using memory tasks and visual memory tasks, but not arithmetic tasks [35]. This might due to the different combination (e.g., mobility and cognitive tasks) used in our study. Moreover, we observed differences between young and older adults in combined dual-task cost of the Timed and Go test (e.g. M = 16.06 and M = 35.57 respectively in subtraction 7), but not in the Four Square Step test (e.g. M = 28.64 and M = 40.22 respectively in subtraction 7). Based on our observations, we conclude that the Four Square Step test requires a more rhythmic cadence compared to the other mobility tasks. This could lead to a higher coordination of the two tasks (motor and cognitive) and might aid in the execution of the task. Nevertheless, we observed that the dual-task cost increased in different age groups, and a decline occurred in the concurrent execution of mobility tasks and cognitive tasks.

Additionally, according to the theoretical models of dual-task interference proposed by Plummer and colleagues [27, 29], we found a mutual interference in both tasks in all groups suggesting an inadequate attentional resources to maintain performance of cognitive and mobility task in dual-task condition compared with each single task. Interestingly, our results showed a greater mutual interference in older groups compared with the other groups, suggesting a greater difficulty in older adults to successfully manage the concurrent tasks.

The higher dual-task cost observed in older adults might reflect the inability to share resources between the mobility and the cognitive tasks [45]. These findings confirm the idea that, in simultaneously performed tasks, the attentional resources must be divided to carry out both primary and secondary task correctly [3, 45].

Our results add a new understanding about the simultaneous performance of mobility and cognitive tasks in different ages. In particular, the results describe the difficulty increasing to manage mobility and cognitive tasks in older people. These results can be useful to better understand the request of attention resources during dual-task performance of older adults and the ability to allocate the attention resources both to the mobility and cognitive tasks. Moreover, our results suggest that future intervention studies, aimed to promote dual-task ability in older people, should assess both mobility and cognitive dual-task cost due to dual-task interference as the result of the interaction between two tasks [29].

Some limitations should be noted. The quantification of mobility performance was characterized by a single parameter (time), and the cognitive tasks used in the present study were arithmetical tasks only. Other spatiotemporal parameters and cognitive tasks should be included in future studies. Due to the cross-sectional nature of the study, a causal relationship between the age of participants and the observed findings regarding mobility measured under single and dual-task conditions cannot be drawn. Longitudinal studies are needed to detect the factors, such as physical and cognitive decline, that may more directly contribute to the decline in dual-task performance.

Despite these limitations, it is important to note that secondary tasks have a destabilizing effect on mobility performance. With aging, the ability to flexibly manage two concurrent tasks may be a challenge. The present study demonstrates that the simultaneous performance of mobility and cognitive tasks might affect the mobility in older adults, an essential skill for the independence and successful performance of everyday activities. Future studies are needed to better understand the dual-task performance in similar and common mobility tasks of activities of daily life and to investigate dual-task effects in relation to different cognitive tasks (e.g., visuomotor processing, reaction time, verbal fluency and decision-making tasks). In everyday life, a mobility performance is rarely conducted alone; instead, it is more often associated with different cognitive activities. Because this simultaneous activity may negatively affect the motor pattern in older adults, we believe that the data from this study may have functional implications for developing future ad hoc preventive intervention programs to minimize difficulties in mobility dual-task performance.

## Supporting information

S1 File Data from participants in this study. (XLS)

## References

[1] Shumway-Cook A, Woollacott M. Motor Control: Translating Research Into Clinical Practice Fourth Edition. Baltimore, MD: Wolters Kluwer Health/Lippincott Williams & Wilkins; 2012.

[2] Bock O. Dual-task costs while walking increase in old age for some, but not for other tasks: an experimental study of healthy young and elderly persons. J Neuroeng Rehabil. 2008;5:27 doi: 10.1186/1743-0003-5-27 ; 19014544 10.1186/1743-0003-5-27 PMC2596160

[3] Woollacott M, Shumway-Cook A. Attention and the control of posture and gait: a review of an emerging area of research. Gait Posture. 2002;16(1):1–14. doi: 10.1016/S0966-6362(01)00156-4 . 12127181 10.1016/s0966-6362(01)00156-4

[4] Chodzko-Zajko WJ, Proctor DN, Fiatarone Singh MA, Minson CT, Nigg CR, Salem GJ, et al American College of Sports Medicine position stand. Exercise and physical activity for older adults. Med Sci Sports Exerc. 2009;41(7):1510–30. Epub 2009/06/12. . 19516148 10.1249/MSS.0b013e3181a0c95c

[5] Magistro D, Candela F, Brustio PR, Liubicich ME, Rabaglietti E. A Longitudinal Study on the Relationship Between Aerobic Endurance and Lower Body Strength in Italian Sedentary Older Adults. J Aging Phys Act. 2015;23(3):444–51. Epub 2014/10/25. doi: 10.1123/japa.2013-0215 . 25341375 10.1123/japa.2013-0215

[6] Brustio PR, Magistro D, Liubicich ME. Changes in temporal parameters during performance of the Step Test in older adults. Gait Posture. 2014;4(1):217–21. doi: 10.1016/j.gaitpost.2014.10.006 . 25455210 10.1016/j.gaitpost.2014.10.006

[7] Park HL, O'Connell JE, Thomson RG. A systematic review of cognitive decline in the general elderly population. Int J Geriatr Psychiatry. 2003;18(12):1121–34. Epub 2003/12/17. doi: 10.1002/gps.1023 . 14677145 10.1002/gps.1023

[8] Yogev-Seligmann G, Hausdorff JM, Giladi N. The role of executive function and attention in gait. Mov Disord. 2008;23(3):329–42. doi: 10.1002/mds.21720 ; 18058946 10.1002/mds.21720 PMC2535903

[9] Holtzer R, Wang C, Verghese J. The relationship between attention and gait in aging: facts and fallacies. Motor Control. 2012;16(1):64–80. doi: 10.1037/0894-4105.21.5.540 ; 22402221 10.1123/mcj.16.1.64 PMC3471155

[10] Hall CD, Echt KV, Wolf SL, Rogers WA. Cognitive and motor mechanisms underlying older adults' ability to divide attention while walking. Phys Ther. 2011;91(7):1039–50. doi: 10.2522/ptj.20100114 . 21527384 10.2522/ptj.20100114

[11] Srygley JM, Mirelman A, Herman T, Giladi N, Hausdorff JM. When does walking alter thinking? Age and task associated findings. Brain Res. 2009;1253:92–9. ; 19084511 10.1016/j.brainres.2008.11.067 PMC2631095

[12] Riediger M, Li S, Lindenberger U. Selection, Optimizazion, and Compensation as Developmental Mechanisms af Adaptive Resource Allocation: Review and Preview In: Birren JE, Schaie KW, editors. Handbook of the Psychology of Aging: Elsevier Science; 2011 p. 289–313.

[13] Pothier K, Benguigui N, Kulpa R, Chavoix C. Multiple Object Tracking While Walking: Similarities and Differences Between Young, Young-Old, and Old-Old Adults. J Gerontol B Psychol Sci Soc Sci. 2015;70(6):840–9. doi: 10.1093/geronb/gbu047 . 24859224 10.1093/geronb/gbu047

[14] Al-Yahya E, Dawes H, Smith L, Dennis A, Howells K, Cockburn J. Cognitive motor interference while walking: a systematic review and meta-analysis. Neurosci Biobehav Rev. 2011;35(3):715–28. Epub 2010/09/14. doi: 10.1016/j.neubiorev.2010.08.008 . 20833198 10.1016/j.neubiorev.2010.08.008

[15] Chu YH, Tang PF, Peng YC, Chen HY. Meta-analysis of type and complexity of a secondary task during walking on the prediction of elderly falls. Geriatr Gerontol Int. 2013;13(2):289–97. Epub 2012/06/15. doi: 10.1111/j.1447-0594.2012.00893.x . 22694365 10.1111/j.1447-0594.2012.00893.x

[16] Doumas M, Krampe RT. Ecological Relevance Determines Task Priority in Older Adults' Multitasking. J Gerontol B Psychol Sci Soc Sci. 2013 doi: 10.1093/geronb/gbt105 . 24149518 10.1093/geronb/gbt105

[17] Nordin E, Moe-Nilssen R, Ramnemark A, Lundin-Olsson L. Changes in step-width during dual-task walking predicts falls. Gait Posture. 2010;32(1):92–7. doi: 10.1016/j.gaitpost.2010.03.012 . 20399100 10.1016/j.gaitpost.2010.03.012

[18] Buchman AS, Boyle PA, Leurgans SE, Barnes LL, Bennett DA. Cognitive function is associated with the development of mobility impairments in community-dwelling elders. Am J Geriatr Psychiatry. 2011;19(6):571–80. doi: 10.1097/JGP.0b013e3181ef7a2e ; 21606900 10.1097/JGP.0b013e3181ef7a2e PMC3101472

[19] Montero-Odasso M, Hachinski V. Preludes to brain failure: executive dysfunction and gait disturbances. Neurol Sci. 2014;35(4):601–4. doi: 10.1007/s10072-013-1613-4 . 24366243 10.1007/s10072-013-1613-4

[20] Boisgontier MP, Beets IA, Duysens J, Nieuwboer A, Krampe RT, Swinnen SP. Age-related differences in attentional cost associated with postural dual tasks: Increased recruitment of generic cognitive resources in older adults. Neurosci Biobehav Rev. 2013;37(8):1824–37. doi: 10.1016/j.neubiorev.2013.07.014 . 23911924 10.1016/j.neubiorev.2013.07.014

[21] Hobert MA, Niebler R, Meyer SI, Brockmann K, Becker C, Huber H, et al Poor trail making test performance is directly associated with altered dual task prioritization in the elderly—baseline results from the TREND study. PLoS One. 2011;6(11):e27831 doi: 10.1371/journal.pone.0027831 ; 22114705 10.1371/journal.pone.0027831 PMC3218043

[22] Hollman JH, Kovash FM, Kubik JJ, Linbo RA. Age-related differences in spatiotemporal markers of gait stability during dual task walking. Gait Posture. 2007;26(1):113–9. doi: 10.1016/j.gaitpost.2006.08.005 . 16959488 10.1016/j.gaitpost.2006.08.005

[23] Hausdorff JM, Schweiger A, Herman T, Yogev-Seligmann G, Giladi N. Dual-task decrements in gait: contributing factors among healthy older adults. J Gerontol A Biol Sci Med Sci. 2008;63(12):1335–43. doi: 10.1093/gerona/63.12.1335 ; 19126846 10.1093/gerona/63.12.1335 PMC3181497

[24] Priest AW, Salamon KB, Hollman JH. Age-related differences in dual task walking: a cross sectional study. J Neuroeng Rehabil. 2008;5:29 doi: 10.1186/1743-0003-5-29 ; 19014583 10.1186/1743-0003-5-29 PMC2607296

[25] MacAulay RK, Brouillette RM, Foil HC, Bruce-Keller AJ, Keller JN. A longitudinal study on dual-tasking effects on gait: cognitive change predicts gait variance in the elderly. PLoS One. 2014;9(6):e99436 doi: 10.1371/journal.pone.0099436 . 24905590 10.1371/journal.pone.0099436 PMC4048284

[26] Seidler RD, Bernard JA, Burutolu TB, Fling BW, Gordon MT, Gwin JT, et al Motor control and aging: links to age-related brain structural, functional, and biochemical effects. Neurosci Biobehav Rev. 2010;34(5):721–33. Epub 2009/10/24. doi: 10.1016/j.neubiorev.2009.10.005 ; 19850077 10.1016/j.neubiorev.2009.10.005 PMC2838968

[27] Plummer P, Eskes G, Wallace S, Giuffrida C, Fraas M, Campbell G, et al Cognitive-motor interference during functional mobility after stroke: state of the science and implications for future research. Arch Phys Med Rehabil. 2013;94(12):2565–74 e6. doi: 10.1016/j.apmr.2013.08.002 ; 23973751 10.1016/j.apmr.2013.08.002 PMC3842379

[28] Smith E, Cusack T, Blake C. The effect of a dual task on gait speed in community dwelling older adults: A systematic review and meta-analysis. Gait Posture. 2016;44:250–8. Epub 2016/03/24. doi: 10.1016/j.gaitpost.2015.12.017 . 27004667 10.1016/j.gaitpost.2015.12.017

[29] Plummer P, Eskes G. Measuring treatment effects on dual-task performance: a framework for research and clinical practice. Frontiers in human neuroscience. 2015;9 doi: 10.3389/fnhum.2015.00225 . 25972801 10.3389/fnhum.2015.00225 PMC4412054

[30] Jansen RJ, van Egmond R, de Ridder H. Task Prioritization in Dual-Tasking: Instructions versus Preferences. PLoS One. 2016;11(7):e0158511 doi: 10.1371/journal.pone.0158511 . 27391779 10.1371/journal.pone.0158511 PMC4938591

[31] Yogev-Seligmann G, Hausdorff JM, Giladi N. Do we always prioritize balance when walking? Towards an integrated model of task prioritization. Mov Disord. 2012;27(6):765–70. doi: 10.1002/mds.24963 . 22419512 10.1002/mds.24963

[32] Muhaidat J, Kerr A, Evans JJ, Skelton DA. Exploring gait-related dual task tests in community-dwelling fallers and non-faller: A pilot study. Physiother Theory Pract. 2012 Epub 2013/01/08. doi: 10.3109/09593985.2012.752056 . 23289962 10.3109/09593985.2012.752056

[33] Muhaidat J, Kerr A, Evans JJ, Pilling M, Skelton DA. Validity of Simple Gait-Related Dual-Task Tests in Predicting Falls in Community-Dwelling Older Adults. Arch Phys Med Rehabil. 2013;95(1):58–64. doi: 10.1016/j.apmr.2013.07.027 . 24071555 10.1016/j.apmr.2013.07.027

[34] Lindenberger U, Marsiske M, Baltes PB. Memorizing while walking: increase in dual-task costs from young adulthood to old age. Psychol Aging. 2000;15(3):417–36. doi: 10.1037/0882-7974.15.3.417 . 11014706 10.1037//0882-7974.15.3.417

[35] Beurskens R, Bock O. Age-related deficits of dual-task walking: a review. Neural Plast. 2012;2012:131608 Epub 2012/08/01. doi: 10.1155/2012/131608 ; 22848845 10.1155/2012/131608 PMC3403123

[36] Wolf SL, Catlin PA, Gage K, Gurucharri K, Robertson R, Stephen K. Establishing the reliability and validity of measurements of walking time using the Emory Functional Ambulation Profile. Phys Ther. 1999;79(12):1122–33. . 10630281

[37] Podsiadlo D, Richardson S. The timed "Up & Go": a test of basic functional mobility for frail elderly persons. J Am Geriatr Soc. 1991;39(2):142–8. Epub 1991/02/01. doi: 10.1111/j.1532-5415.1991.tb01616.x . 1991946 10.1111/j.1532-5415.1991.tb01616.x

[38] Dite W, Temple VA. A clinical test of stepping and change of direction to identify multiple falling older adults. Arch Phys Med Rehabil. 2002;83(11):1566–71. Epub 2002/11/08. . 12422327 10.1053/apmr.2002.35469

[39] Steffen TM, Hacker TA, Mollinger L. Age- and gender-related test performance in community-dwelling elderly people: Six-Minute Walk Test, Berg Balance Scale, Timed Up & Go Test, and gait speeds. Phys Ther. 2002;82(2):128–37. . 11856064 10.1093/ptj/82.2.128

[40] Montero-Odasso M, Bergman H, Phillips NA, Wong CH, Sourial N, Chertkow H. Dual-tasking and gait in people with mild cognitive impairment. The effect of working memory. BMC Geriatr. 2009;9:41 doi: 10.1186/1471-2318-9-41 ; 19723315 10.1186/1471-2318-9-41 PMC2748075

[41] Granacher U, Bridenbaugh SA, Muehlbauer T, Wehrle A, Kressig RW. Age-related effects on postural control under multi-task conditions. Gerontology. 2011;57(3):247–55. doi: 10.1159/000322196 . 20980734 10.1159/000322196

[42] Yang L, He C, Pang MYC. Reliability and Validity of Dual-Task Mobility Assessments in People with Chronic Stroke. PLoS One. 2016;11(1):e0147833 doi: 10.1371/journal.pone.0147833 . 26808662 10.1371/journal.pone.0147833 PMC4726712

[43] Beauchet O, Dubost V, Aminian K, Gonthier R, Kressig RW. Dual-task-related gait changes in the elderly: does the type of cognitive task matter? J Mot Behav. 2005;37(4):259–64. doi: 10.1159/000081435 . 15967751

[44] Brustio PR, Magistro D, Rabaglietti E, Liubicich ME. Age-related differences in dual task performance: A cross-sectional study on women. Geriatr Gerontol Int. 2017;17(2):315–21. doi: 10.1111/ggi.12700 . 26712164 10.1111/ggi.12700

[45] Bernard-Demanze L, Dumitrescu M, Jimeno P, Borel L, Lacour M. Age-related changes in posture control are differentially affected by postural and cognitive task complexity. Curr Aging Sci. 2009;2(2):139–49. doi: 10.2174/1874609810902020135 . 20021408

